# Anterior relapse or posterior drift after intraoral vertical ramus osteotomy

**DOI:** 10.1038/s41598-020-60838-1

**Published:** 2020-03-02

**Authors:** Satoshi Rokutanda, Shin-Ichi Yamada, Souichi Yanamoto, Hiroshi Sakamoto, Kohei Furukawa, Hiromi Rokutanda, Tomoko Yoshimi, Takuya Nakamura, Yukiko Morita, Noriaki Yoshida, Masahiro Umeda

**Affiliations:** 1 Department of Clinical Oral Oncology, Nagasaki University Graduate School of Biomedical Sciences, 1-7-1 Sakamoto, Nagasaki City, Nagasaki 852-8588 Japan; 2 Department of Oral and Maxillofacial Surgery, Juko Memorial Nagasaki Hospital, 1-73 Akunoura Town, Nagasaki city, Nagasaki 850-0063 Japan; 3 Department of Dentistry and Oral Surgery, Shinshu University School of Medicine, 3-1-1 Asahi, Matumoto City, Nagano 390-8621 Japan; 4 Department of Orthodontics and Dentofacial Orthopedics, Nagasaki University Graduate School of Biomedical Sciences, 1-7-1 Sakamoto, Nagasaki City, Nagasaki 852-8588 Japan

**Keywords:** Oral diseases, Outcomes research

## Abstract

This study aimed to evaluate the factors contributing to postoperative anterior relapse or posterior drift of the distal segment after intraoral vertical ramus osteotomy. A retrospective cohort study was conducted which included 31 patients who underwent setback surgery for mandibular prognathism by the intraoral vertical ramus osteotomy technique. Uni- and multivariate analyses were performed to determine the association of potential explanatory variables (sex, age, magnitude of setback, differences in setback magnitude between sides (right/left), duration of splint use, Angle’s classification of malocclusion, mandibular angle, and tightness of occlusion of the molars) with positional changes in the distal segment. The setback magnitude was only significant factor affecting (P = 0.015) for posterior drift, with significant posterior in setback magnitudes of less than 7.25 mm. Posterior drift after intraoral vertical ramus osteotomy is less likely if setback magnitude exceeds 7.25 mm. For setbacks less than 7.25 mm, posterior drift should either be carefully corrected postoperatively, or an alternative surgical technique should be used. The setback magnitude showed a significant association with the risk of posterior drift following intraoral vertical ramus osteotomy, and the determined cut-off value may serve as a predictor for postoperative outcomes.

## Introduction

Intraoral vertical ramus osteotomy (IVRO) is a common orthognathic surgical procedure in patients with skeletal mandibular prognathism. This mandibular setback procedure is believed to have several advantages, including ease of performance and a low risk of mandibular nerve paraesthesia^1^. However, as the split segments are not fixed, IVRO is inferior to sagittal split ramus osteotomy in terms of postoperative stability.

Previous studies on skeletal stability after mandibular setback surgery using the IVRO technique have reported both anterior relapse and posterior drift of the mandible. Choi *et al*.^2,3^, Jung *et al*.^4^, and Greebe and Tuinzing^5^ reported that posterior mandibular drift is more common after IVRO. However, Kung and Leung^6^, Halvorsen *et al*.^7^, Chen *et al*.^8^, Nihara *et al*.^9^, and Ahlen *et al*.^10^ have reported anterior relapse after IVRO. Conversely, Ayoub *et al*.^11^ reported almost no anterior relapse or posterior drift after IVRO. In view of these conflicting reports, the direction of mandibular displacement after IVRO remains unclear. Regarding the relationship between the setback magnitude after IVRO and the accompanying skeletal change, reports show a decrease in the latter with an increase in the setback magnitude^2–4,8,12^. Conversely, one study has documented that the skeletal change increases as the setback magnitude increases^6^. Furthermore, studies have suggested a multitude of potential factors that can cause this skeletal change, ranging from setback magnitude to other factors such as the tightness of occlusion^13^.

An investigation into the causes for anterior relapse and posterior drift after IVRO is essential, as this knowledge may guide the selection of appropriate surgical and postoperative management techniques to reduce the incidence of these events, which may complicate subsequent orthodontic treatment.

In this study, we investigated whether positional changes in the distal segment after IVRO were due to anterior relapse or posterior drift, and evaluated its association with potential explanatory factors.

## Methods

### Subjects

This study received Institutional Review Board approval (Nagasaki University Graduate School of Biomedical Sciences, approval number 16020826), and all participants provided written informed consent. All experiments were performed in accordance with relevant guidelines and regulations. The study included patients diagnosed with skeletal mandibular prognathism who underwent IVRO between November 2012 and February 2017 at the Unit of Translational Medicine in the Department of Clinical Oral Oncology at the Nagasaki University Graduate School of Biomedical Sciences. Inclusion criteria comprised patients who (1) underwent mandibular setback using IVRO performed by the same surgeon; (2) did not have previous orthodontic or maxillary surgery; and (3) were available for all evaluation time frames. Patients were excluded if they (1) did not agree to participate; (2) underwent bimaxillary surgery; or (3) were lost to follow-up at specific time intervals (1 week before and after the designated follow-up date). We post-operatively evaluated both the left and right sides in 31 patients (19 women and 12 men) using computed tomography (CT) (Aquilion 64^TM^, Canon Medical Systems Corporation, Tochigi, Japan) during follow-up. In “Diagnostic reference levels based on results of the latest domestic fact-finding survey” released in Japan in 2015, the diagnostic reference level (DRL) of an adult simple head-CT scan was determined to be 85 mGy for CTDIvol and 1,350 mGy.cm for DLP. From this, it can be deduced that the average radiation dose per simple head-CT scan is approximately 2.835 mSv. In our department, the tube voltage is fixed at 120kVp, the tube current with auto-exposure control (AEC) uses 3-mm slices for soft tissues, photographs are obtained with an attached quantum filter, and the actual tube current varies from 32 to 100 mA, for the maximal reduction in radiation exposure from CT in jaw deformity patients. The mean CTDIvol of 12.6 mGy and mean DLP of 191 mGy.cm is obtained by using these methods. Hence, mean radiation exposure per CT calculated from the DLP value would be 0.401 mSv, obtained by using a dose-conversion coefficient of 0.0021. Consequently, in our department, CT scans are designed such that jaw deformity patients receive only approximately 15% of the typical dose from a simple head-CT scan, which was considered while performing the multiple CT scans.

Three-dimensional (3D) CT images were used to evaluate the anterior relapse or posterior drift of the distal segment. CT was performed preoperatively and immediately after surgery to check for complications; subsequent postoperative evaluation was performed at 1, 3, 6, and 12 months. The CT images were acquired with the mouth closed to ensure reproducibility of the position of the distal segment during measurements.

All statistical analyses were performed with SPSS software (version 24.0; Japan IBM Co., Tokyo, Japan). A P-value less than 0.05 was considered statistically significant.

### Surgical procedure and IMF

The surgical procedure of IVRO was performed under general anaesthesia. An intraoral incision was made at the anterior border of ramus. A pair of Bauer retractors was placed in the sigmoid and antegonial notches to visualize the ante-lingular prominence and to prevent bleeding from the internal maxillary artery, and a subcondylar osteotomy was performed using an oscillating saw. The distal fragment was slid distally and was placed medial to the proximal fragments. The distal fragment of the mandible was placed in the planned postsurgical position and was stabilized using rigid IMF with a splint to the maxillary dental arch.

### Assessment methods

#### Measurement of anterior relapse or posterior drift of the distal segment, and positional changes in distal segments by setback magnitude after IVRO

**Figure 1:**
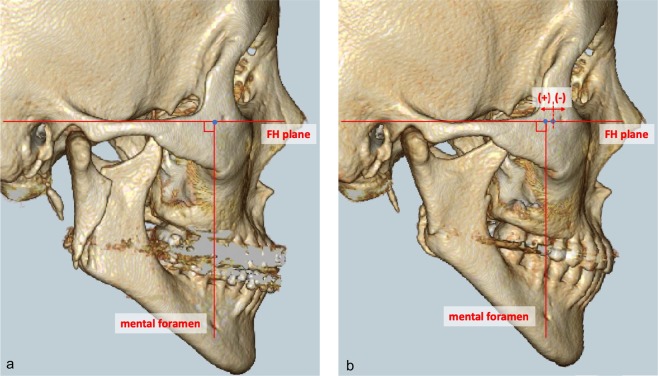
Measurement of anterior relapse or posterior drift of distal segment and positional changes in distal segments by setback magnitude after IVRO. (**a**) Immediately after IVRO (**b**) 12 months after IVRO. IVRO: Intraoral vertical ramus osteotomy.

We measured the positional changes in the distal segment after IVRO over time, on the CT images. The Frankfurt plane was delineated on the 3D CT images on the left and right sides. A perpendicular line was drawn from the centre of the mental foramen toward the Frankfurt plane. The length from the upper end of the external acoustic meatus to the point of intersection with the perpendicular line was measured at each session. The measurement immediately after surgery was 0 mm, and subsequent differences at each session were recorded. In addition, we classified and compared the anterior relapse or posterior drift after IVRO according to the magnitude of setback. We denoted anterior relapse as negative (−) and posterior drift as positive (+) (Fig. 1a,b). All measurements were performed twice by three oral surgeons. Two or more oral surgeons were present during the measurements to ensure the accuracy of the measurements. All obtained measurements were integrated and averaged.

#### Univariate and multivariate analyses of factors possibly related to anterior relapse or posterior drift of the distal segment after IVRO

Univariate and multivariate analyses were performed using displacement of the distal segment (anterior relapse or posterior drift) after IVRO as the dependent variable, and the potential explanatory variables as independent variables. The explanatory variables included sex, age, magnitude of setback, differences in setback magnitude between sides (left/right), duration of splint use, Angle’s classification of malocclusion, occlusion tightness, and angle of mandible (high, normal, or low). The period of use of the splint was basically 3 months, but the orthodontist analysed the occlusion after orthodontic treatment for approximately 30 minutes in a horizontal position. At the time, if the occlusion was maintained in the desired position while standing, the splint was removed. Occlusion tightness was classified by the presence or absence of occlusal contacts between bilateral first and second molars after IVRO; those with occlusal contact were designated as group 1, while those with occlusal contact between at least 1 set of molars were designated as group 2. The angle of the mandible was categorised as high, normal, or low according to the criteria proposed by Nezu *et al*.^14^. The occlusal contacts between the first and second molars and the angle of the mandible were evaluated twice by 4 orthodontists on separate occasions. Profit *et al*.^15^ reported that mandibular relapses exceeding 2 mm after orthognathic surgery are clinically significant. Therefore, any anterior or posterior displacement of the distal segment after IVRO was considered to be anterior relapse or posterior drift, respectively, only if the magnitude was 2 mm or greater. For factors found to be significantly associated with anterior relapse or posterior drift during multivariate analysis, cut-off values were determined from the receiver operating characteristic (ROC) curve.

## Results

The mean age of the cohort at the time of surgery was 27.0 (range: 17 to 54) years; the cohort comprised 12 male and 19 female patients. The magnitude of setback achieved with IVRO ranged from 0 to 13 mm (with a mean of 6.4 mm). The setback magnitude between the left and right sides was considered to be different if the discrepancy was 2 mm or more; this was noted in 15 cases. The duration of splint use ranged from 0 to 6 months, with an average of 1.7 months. Angle’s classification of malocclusion after IVRO was defined as class I for 35 sides, and class II for 27 sides. The mandibular angle pre-IVRO was categorised as high, normal, and low, in 14, 15, and 2 sides, respectively. The mandibular angle post-IVRO was categorised as high, normal, and low, in 16, 14, and 1 side(s), respectively. Occlusion tightness was observed in 28 cases for Group 1 and 3 cases for Group 2. (Table 1).

**Table 1:** Demographic and clinical characteristics of the study patients.

Case number	Sex	Age (years)	Magnitude of setback (mm) on left side	Magnitude of setback (mm) on right side	Difference in setback magnitude between left and right sides ≥ 2 mm	Duration of splint use (months)	Angle’s classification (left/right)	Classification of mandibular angle (high, low, normal), (pre-/post-operative)	Occlusion tightness Group1:1 Group2:2
1	M	45	5	5	No	1	I/I	H/N	1
2	M	19	9.5	7.5	Yes	4	I/I	H/H	1
3	M	23	5	5	No	0	I/I	L/N	1
4	F	26	6.5	10.5	Yes	1	II/I	N/N	1
5	F	24	3	3	No	3	II/II	H/H	1
6	F	21	4	7	Yes	3	I/I	N/H	1
7	M	21	6	9	Yes	2	II/II	H/H	1
8	F	28	12	12	No	0	I/I	H/H	1
9	M	21	11	11	No	1	II/II	H/H	1
10	F	20	8	8	No	1	I/I	N/H	1
11	F	45	0	7	Yes	3	II/I	N/N	2
12	F	51	5	7	Yes	1	II/II	N/N	1
13	F	25	3.5	3.5	No	2	II/II	H/H	1
14	F	17	3	4	No	2	II/II	H/H	2
15	F	39	5.5	8	Yes	1.5	I/II	N/N	1
16	F	18	0.8	5.2	Yes	1	II/II	N/H	1
17	M	19	6.5	6.5	No	1.5	II/II	L/H	1
18	M	48	6	2	Yes	3	II/II	H/H	1
19	F	20	13	13	No	6	II/II	N/N	1
20	M	19	6.5	5	No	1	I/I	N/N	1
21	M	42	3	1	Yes	1	I/I	N/L	1
22	F	19	9	9	No	1	I/I	H/N	1
23	F	18	10	8	Yes	2	I/I	H/H	2
24	F	26	8	8	No	3	I/I	H/H	1
25	F	24	2	3	No	1	I/I	N/N	1
26	M	17	9.5	5.5	Yes	0.5	I/I	H/N	1
27	M	54	2	6	Yes	1	I/I	N/H	1
28	F	18	7.5	7.5	No	0.5	II/II	N/N	1
29	F	25	2	7.5	Yes	1	I/I	H/H	1
30	F	24	0	3	Yes	2	I/I	N/N	1
31	M	20	11	11	No	1.5	II/II	N/N	1
Total	M:12 F:19	27.0	6.4	6.4	Yes:15 No:16	1.7	I:35 II:27	Pre-operative (High: 14, Normal: 15, Low: 2	Grup1:28 Group2:3
								Post-operative (High: 16, Normal: 14, Low: 1)	

M: Male, F: Female, I: Angle Class I, II: Angle Class II, H: High, L: Low, N: Normal, occlusal contacts were designated: group 1, occlusal contacts between at least 1 set of molars were designated:group 2.

### Positional changes in the distal segment after IVRO in the entire cohort and according to magnitude of setback

The setback magnitude was measured after reversion of the distal segment by IVRO. The magnitude of anterior relapse or posterior drift after IVRO ranged from −4.1 mm to 9.5 mm. After IVRO, the distal segment tended to drift posteriorly by an average of 2.2 mm. Overall (n = 62), the posterior drift of the distal segment at 1 month after surgery was 2.6 ± 3.7 mm. This magnitude showed an initial increase over time, before a reduction at the 1-year point, with a mean value of 2.1 ± 3.0 mm at 1 year. The posterior drift was significant across all time points.

**Figure 2:**
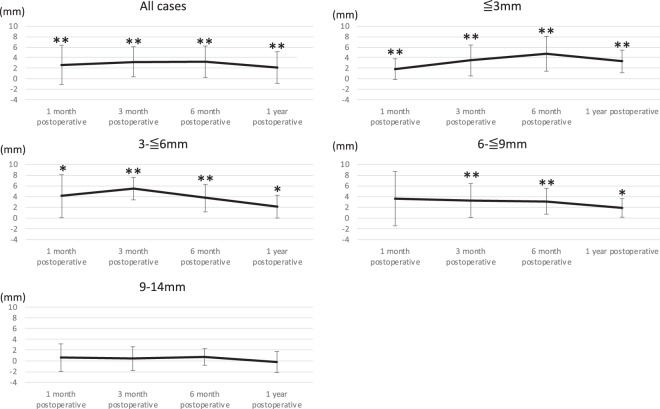
Positional changes in the distal segment after IVRO in the entire cohort and according to setback magnitude. IVRO: Intraoral vertical ramus osteotomy.

In cases in which the magnitude of setback was 0 to ≤ 3 mm (n = 14), the posterior drift of the distal segment at 1 month after surgery was 1.8 ± 2.0 mm. This increased up to 6 months after surgery, with a mean value of 3.3 ± 2.1 mm at 1 year. Significant differences were observed at each time point. In cases in which the magnitude of setback was 3 to ≤ 6 mm (n = 16), the posterior drift of the distal segment at 1 month after surgery was 4.0 ± 4.0 mm. This increased at 3 months after surgery, and the mean was 2.1 ± 2.1 mm at 1 year. Significant differences were observed across all time points. In cases in which the magnitude of setback was 6 to ≤ 9 mm (n = 22), the posterior drift of the distal segment at 1 month after surgery was 3.6 ± 5.1 mm. This decreased over time, with a mean value of 1.9 ± 1.7 mm at 1 year. A significant difference was observed at each time point, except for 1 month after surgery. In cases in which the magnitude of setback was >9 mm (n = 10), the posterior drift of the distal segment at 1 month after surgery was 0.6 ± 2.6 mm. This displacement was negligible, with a mean value of −0.2 ± 2.0 mm at 1 year after surgery. No significant differences were observed at the assessed time points (Table 2, Fig. 2). The standard deviation of the results was very oscillating, and even if cases in which the difference in setback from left to right was 4.0 mm or more were excluded, no improvement in the oscillating standard deviation was observed.

**Table 2:** Positional changes in the distal segments after IVRO and positional changes in the distal segments according to setback magnitude.

All cases (n = 62)					
	Immediately after IVRO	1 month postoperatively	3 months postoperatively	6 months postoperatively	1 year postoperatively
Average	0	2.6	3.2	3.2	2.1
SD		3.7	2.9	3.0	3.0
P-value		2.7 × 10^−5^**	6.9 × 10^−10^**	6.2 × 10^−9**^	9.7 × 10^−5^**
**0 to ≤ 3** **mm (n = 14)**					
Average	0	1.8	3.5	4.8	3.3
SD		2.0	2.9	3.3	2.1
P-value		7.8 × 10^−3^**	6.6 × 10^−5^**	5.8 × 10^−4^**	5.4 × 10^−4^**
**3 to ≤ 6 mm (n = 16)**					
Average	0	4.0	5.5	3.7	2.1
SD		4.0	2.1	2.5	2.1
P-value		2.0 × 10^−2^*	6.2 × 10^−6^**	2.1 × 10^−3^**	2.4 × 10^−2^*
**6 to ≤ 9 mm (n = 22)**					
Average	0	3.6	3.3	3.2	1.9
SD		5.1	3.2	2.4	1.7
P-value		6.0 × 10^−2^	4.1 × 10^−3^**	2.1 × 10^−3^**	2.4 × 10^−2^*
**>9 mm (n = 10)**					
Average	0	0.6	0.4	0.7	−0.2
SD		2.6	2.2	1.6	2
P-value		0.51	0.71	0.25	0.87

IVRO: Intraoral vertical ramus osteotomy, SD: Standard deviation.

**Figure 3:**
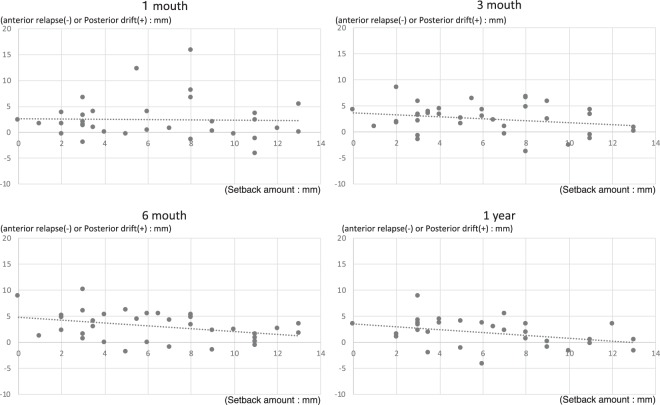
Multiple/linear regression analysis for magnitude of horizontal movement of the mandible (all cases).

Since the magnitude of mandibular retraction is a continuous variable, multiple/linear regression analysis was used to analyse the magnitude of mandibular horizontal movement. Multiple/linear regression analysis showed no change in the magnitude of posterior drift due to the magnitude of distal setback, one month after surgery. However, at 3 months, 6 months, and 1 year after IVRO, the degree of posterior drift showed a tendency to decrease as the setback magnitude increased (Fig. 3).

**Figure 4:**
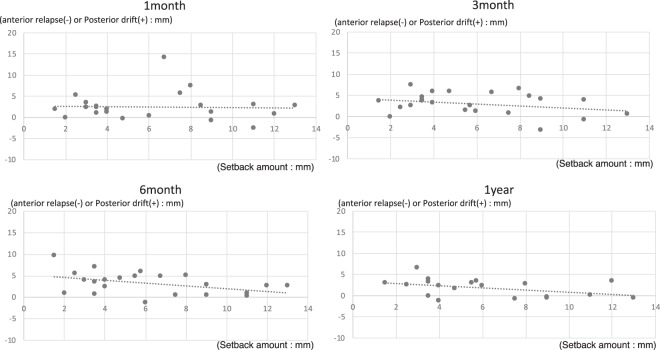
Multiple/linear regression analysis for magnitude of horizontal movement of the mandible (all cases: average).

**Figure 5:**
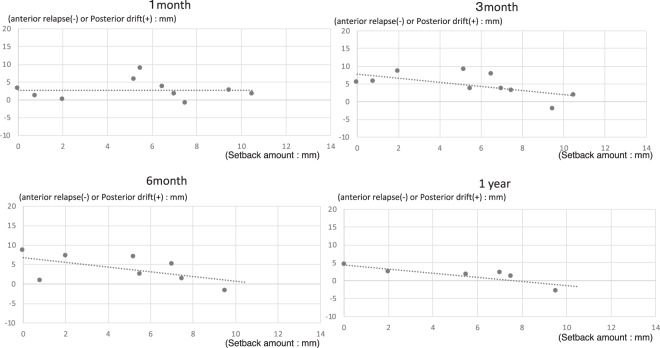
Multiple/linear regression analysis for magnitude of horizontal movement of the mandible (cases wherein the magnitude of setback is different bilaterally).

In addition, multiple/linear regression analysis was performed for all cases using the average value of the magnitude of setback for each case and of the antero-posterior movement of the mandible, when the magnitude of setback was different bilaterally. As observed in multiple/linear regression analysis of all cases, multiple/linear regression analysis showed no change in the magnitude of posterior drift due to the magnitude of distal setback, one month after surgery. However, at 3 months, 6 months, and 1 year after IVRO, the degree of posterior drift showed a tendency to decrease as the setback magnitude increased (Figs. 4 and 5).

### Univariate and multivariate analyses

Univariate analyses found that sex, age, differences in magnitude of setback between sides (left/right), duration of splint use, occlusion tightness, Angle’s classification of malocclusion, and mandibular angle were not significantly associated with posterior drift of the distal segment after IVRO. The only significantly associated variable was magnitude of setback (P = 0.001) (Table 3). Similarly, in a multiple logistic regression analysis, only the magnitude of setback was found to be significantly associated with posterior drift (P = 0.015) (odds ratio: 0.43) (95% confidence interval: 0.128–0.85) (Table 4).

**Table 3:** Univariate analysis of the displacement of the distal segment and potential explanatory variables.

Potential explanatory variables	P-value
Sex^a^	0.236
Age^a^	0.242
Magnitude of setback^a^	0.001**
Duration of splint use^c^	0.752
Angle’s classification of malocclusion^b^	0.945
Left/right differences in setback magnitude^b^	0.722
Occlusion tightness^c^	0.934
Preoperative: high/low angle^c^	0.524
Postoperative: high/low angle^c^	0.354

^a^Mann-Whitney U test. ^b^Fisher’s exact test. ^c^Wilcoxon rank-sum test.

**Table 4:** Multivariate analysis of the association between displacement of the distal segment and potential explanatory variables.

	P-value	Odds Ratio	95% CI: min	95% CI: max
Sex	0.116	422	0.225	790687.098
Age	0.244	1.115	0.929	1.338
Magnitude of setback	0.015*	0.43	0.218	0.85
Duration of splint use	0.109	5.441	0.686	43.12
Angle’s classification of malocclusion	0.424	0.339	0.024	4.812
Left/right differences in setback magnitude	0.292	1.781	0.608	5.214
Occlusion tightness	0.659	1.656	0.176	15.589
Preoperative: high/low angle	—	—	—	—
Postoperative: high/low angle	—	—	—	—

CI: Confidence interval.

### Calculation of the cut-off value from the ROC curve

**Figure 6:**
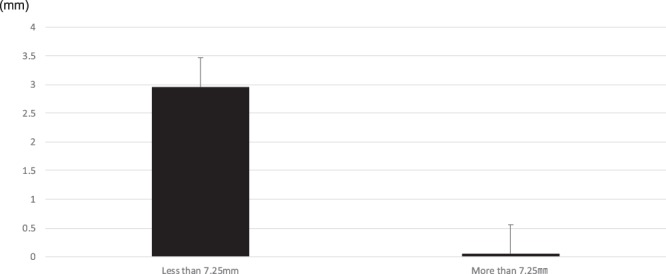
Calculation of cut-off value from the ROC curve. ROC: Receiver operating characteristic.

To quantify the relationship between posterior drift after IVRO and magnitude of setback, a cut-off value was obtained from the ROC curve using the Youden index as an indicator. The findings revealed that posterior drift after IVRO was significantly more likely in cases where the magnitude of setback was 7.25 mm or less, compared to cases where it was more; the corresponding posterior drifts were 0.05 mm and 3.0 mm, respectively (Fig. 6).

## Discussion

In cases of mandibular setback using IVRO, it has been debatable as to what extent anterior relapse or posterior drift occurs postoperatively, and what factors may be associated with their occurrence. This is pertinent, as considerable magnitudes of relapse require extensive compensatory orthodontic treatment after surgery. In fact, Profit *et al*.^15^ stated that more than 2.0 mm of postoperative displacement reflects poor outcomes and may lead to prolonged periods of postoperative orthodontic treatment with sub-optimal occlusion. Therefore, the present study aimed to investigate the skeletal changes in the mandible after IVRO, and evaluated the factors that possibly affect subsequent postoperative anterior relapse or posterior drift.

In this study, posterior drift was the major skeletal change observed after IVRO. Furthermore, the degree of posterior drift decreased as the setback magnitude increased. When the magnitude of setback is large, there is no statistically significant difference in the magnitude of posterior drift 1 year after IVRO. In addition, there was a clear tendency for anterior relapse when the setback magnitude was 9 mm or more. Neither sex, age, duration of splint use, Angle’s classification of malocclusion, left-right difference in setback magnitude, occlusal tightness, nor mandibular angle were related to posterior drift after IVRO. Indeed, only the magnitude of setback was found to be a significant factor, and ROC curve analysis revealed that values of less than 7.25 mm were more likely to cause significant posterior drifts of 2 mm or more after IVRO. Thus, setback magnitudes below this threshold may likely indicate the need for either postoperative orthodontic treatment, or the consideration of alternative surgical techniques.

These findings may be attributed to the fact that after IVRO, the distal segment is in contact with the soft tissues on the posterior aspect; therefore, as the magnitude of setback increases, posterior drift is reduced. It may therefore be considered that the magnitude of setback by IVRO is limited by the distance between the posterior edge of the ramus and the lingula of the mandible, and by the point where it comes into contact with the soft tissues on the posterior aspect.

This study has some limitations. Firstly, it was a preliminary study involving a small number of patients. Secondly, if the setback magnitude was small, in order to avoid the need for more complex orthodontic treatment due to the large magnitude of posterior drift after IVRO, we considered stricter orthodontic treatment or the selection of other surgical methods. As a result, this limited the number of cases suitable for this study. Thirdly, the CT scan was performed with the splint removed, the patient was instructed to bite in the natural position, and intermaxillary fixation was performed using intermaxillary elastics. Therefore, the vertical changes in mandibular positions could not be measured. Vertical changes may also contribute to mandibular stability and should be considered. Future studies should consider increasing the sample size by including cases from other sites in a multicentre study.

In conclusion, follow-up data after IVRO revealed that the distal segment has a tendency to drift posteriorly, with the magnitude of posterior drift being inversely proportional to the magnitude of setback. Therefore, the possibility of posterior drift needs to be considered in patients undergoing IVRO. When the magnitude of setback is 7.25 mm or less, posterior drift should either be carefully corrected postoperatively, or an alternative surgical technique should be used.

## Data Availability

All data generated or analysed during this study are included in this published article.
